# Effect of Temperatures on Cyclic Fatigue Resistance of 3 Different Ni-Ti Alloy Files

**DOI:** 10.1016/j.identj.2023.06.008

**Published:** 2023-07-07

**Authors:** Sirawut Hiran-us, Sarita Morakul

**Affiliations:** a Department of Operative Dentistry, Faculty of Dentistry, Chulalongkorn University, Bangkok, Thailand; b Composite Structures Research Unit, Department of Mechanical Engineering, Faculty of Engineering, Chulalongkorn University, Bangkok, Thailand

**Keywords:** Austenite, Body temperature, Cyclic fatigue, Martensite, Nickel-titanium

## Abstract

**Objective:**

The aim of this study was to investigate and compare the effect of temperature on the cyclic fatigue resistance of conventional (ProTaper Universal [PTU]), Gold-Wire (ProTaper Gold [PTG]), and Fire-Wire (EdgeTaper Platinum [ETP]) nickel-titanium alloy files.

**Method:**

Twenty files from each system were tested for cyclic fatigue resistance in an artificial canal model. The experiments were performed at room temperature and body temperature in controlled temperature water. Magnified videos were recorded using a dental operating microscope integrated camera during testing to detect file fracture. The number of cycles to failure (NCF) was calculated. The type of failure was investigated macroscopically and microscopically with a dental operating microscope and scanning electron microscope, respectively.

**Result:**

The NCF at room temperature was significantly higher compared with body temperature in each system (*P* < .001). Compared at the same temperature, the ETP group demonstrated the highest NCF, followed by the PTG and PTU groups (*P* < .001). All files demonstrated cyclic fatigue failure macroscopically and microscopically.

**Conclusions:**

The 3 alloy files were affected by temperature. The cyclic fatigue resistance was reduced at the higher temperature and increased at the lower temperature. If the files are geometrically identical, files made of Fire-Wire are preferred compared with Gold-Wire and conventional nickel-titanium alloys based on cyclic fatigue resistance.

## Introduction

Nickel-titanium (NiTi) rotary files have been used for root canal preparation since the early 1990s. These files have excellent properties and characteristics for shaping root canals.^1^, ^2^, ^3^ Their use improved the root canal preparation technique by reducing operator fatigue and procedural errors.^4^ ProTaper Universal (PTU, Dentsply Tulsa Dental Specialties) and ProTaper Gold (PTG, Dentsply Tulsa Dental Specialties) are multiple file systems which comprise shaping (S1 and S2) and finishing (F1, F2, F3, F4, and F5) files. The 2 systems are geometrically identical but differ in the type of alloy they are produced from. PTU is made of conventional NiTi alloy, whilst PTG is made of Gold-Wire alloy. EdgeTaper Platinum (ETP, EdgeEndo) rotary files are produced by a different manufacturer using the same geometrical design and made of Fire-Wire alloy.

Although these files are well suited for root canal preparation, they can break during usage. Cyclic fatigue fracture results from the file rotating in a curved canal^5^ and is affected by many factors, including root canal curvature^6^ and file factors.^7^

One factor influencing a file's cyclic fatigue resistance is the type of NiTi alloy used in manufacturing the file. In 2007, the first thermomechanically treated alloy with superior physical properties became available.^8^ Subsequently, manufacturers have continued developing their own thermomechanically treated alloys to achieve better properties. Numerous studies found that thermomechanical processing NiTi alloys improved their cyclic fatigue resistance from 30% to 900% compared with their original alloys.^8^, ^9^, ^10^, ^11^, ^12^ However, several studies determined that body temperature had a negative effect on the cyclic fatigue resistance of these alloys,^11^^,^^13^, ^14^, ^15^, ^16^, ^17^ whilst other studies did not find a significant effect.^11^^,^^15^^,^^16^ Based on these findings, investigating the efficacy of rotary files in terms of cyclic fatigue resistance at room temperature is no longer acceptable. Furthermore, studies performed at body temperature are still limited. Investigating the effect of temperature on cyclic fatigue resistance in files with differently produced alloys is crucial. The clinicians and manufacturers can choose the appropriate alloy for their clinical use or new rotary file system development, respectively.

This is the first study that investigated and compared the effect of temperature on the cyclic fatigue resistance of NiTi rotary files that shared the same design but were made of Gold-Wire, Fire-Wire, and conventional NiTi alloys, which can show the true effect of the produced alloys. Thus, the purposes of this study were to investigate the effect of body and room temperature on the cyclic fatigue resistance and compare the efficacy of conventional, Gold-Wire, and Fire-Wire NiTi alloy files.

## Method

**Fig. 1:**
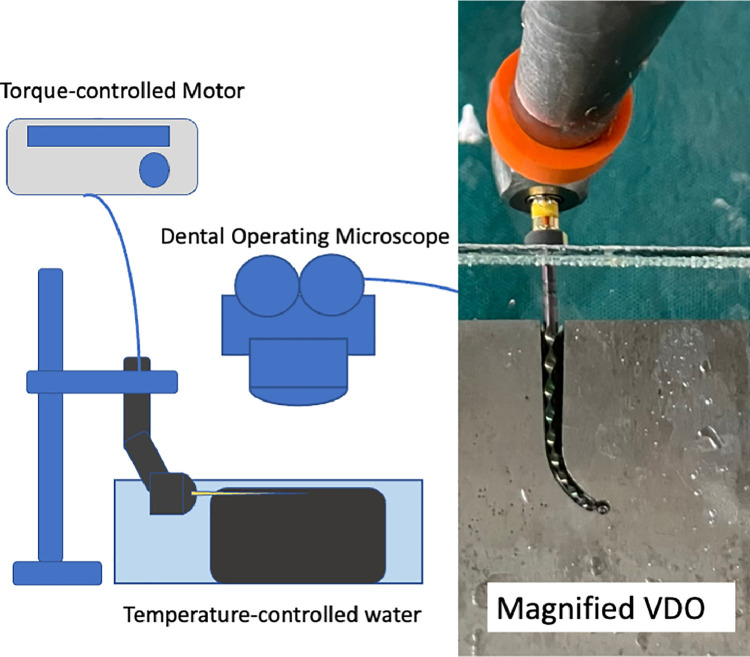
Illustration of the test model. Cyclic fatigue testing was investigated under magnification using a dental operating microscope.

New files from 3 NiTi rotary file systems, PTU, PTG, and EPT, were used in this study as representatives of the conventional, Gold-Wire, and Fire-Wire NiTi alloys, respectively. The sample size calculation was performed with G*Power with a test power of 0.8 (G*Power 3.1.9.4 software for Windows; Franz Faul) using data from our pilot study. This calculation indicated that 2 samples per group was sufficient for statistical analysis to detect a significant difference (Type I error was 0.05); however, the sample size was increased to 10 samples per group to increase the power of the study. Thus, 20 F5 (50/.05 variable tapered) files of each system were examined using a dental operating microscope (OPMI PROERGO, Zeiss, Germany) to confirm that the files had no visible defects. Any file with a defect was discarded and replaced with a new file. The files were tested for cyclic fatigue resistance in a steel artificial canal model that was produced by a milling machine (CNC Milling sister SD543) from a 3D blueprint. The size of the artificial canal resembled the file dimensions (60° curvature and 5-mm radius). The model was set in temperature-controlled distilled water in a glass container. The accuracy of the experimental temperatures was monitored using 2 thermometers that were set at the top and the bottom of the container. Ten files of each system were randomly tested at room temperature (20 ± 1 °C) and body temperature (37 ± 1 °C). The files were rotated at 300 rpm using a torque-control motor (VDW Silver Reciproc motor) as recommended by the manufacturers (3.1 N•cm). Magnified videos were recorded using a dental operating microscope integrated camera during testing to detect file fracture (Figure 1). The number of cycles to failure (NCF) was calculated using the time to fracture in seconds for each sample. The broken files were inspected in the lateral view with a dental operating microscope. Two broken files in each group were randomly selected to investigate the fractured surfaces with a scanning electron microscope (SEM; Quanta). The normal distribution of the data was tested with the Shapiro–Wilk test. The data were statistically analysed using the independent *t* test for comparing the difference between temperatures within the system to investigate the effect of temperature on each alloy. One-way analysis of variance and Fisher least significant difference tests were used to compare the differences between systems at the same temperature to compare the efficacy between systems at the same temperature at a 5% significance level.

## Results

The data were normally distributed (*P* > .05). The mean and standard deviation of the NCF are presented in the Table. The NCF at room temperature was significantly higher compared with body temperature in each system (*P* < .001). Compared at the same temperature, the ETP group demonstrated the highest NCF followed by the PTG and PTU groups. The differences between rotary systems were significant (*P* < .001).

**Table:** Descriptive statistical data of the number of cycles to failure of the ETP, PTG, and PTU files at room and body temperature.

	Room temperature (20 ± 1 °C)				Body temperature (37 ± 1 °C)			
	Mean ± SD	95% CI	Min–max	Median	Mean ± SD	95% CI	Min–max	Median
ETP	1811.00 ± 580.73^aA^	1799–1822	975–2865	1842.5	1047.00 ± 219.24^aB^	1043–1051	775–1470	1022.5
PTG	832.00 ± 116.89^bA^	829–834	660–1095	807.5	409.00 ± 117.04^bB^	406–411	210–575	412.5
PTU	169.00 ± 36.82^cA^	167–169	100–210	170.0	94.5.00 ± 12.428^cB^	94.25–94.75	75–110	95.0

ETP, EdgeTaper Platinum; PTG, ProTaperGold; PTU, ProTaper Universal.

Columns: Different lowercase letters indicate a significant difference in the same column (*P* < .001).

Rows: Different uppercase letters indicate a significant difference in the same row (*P* < .001).

**Fig. 2:**
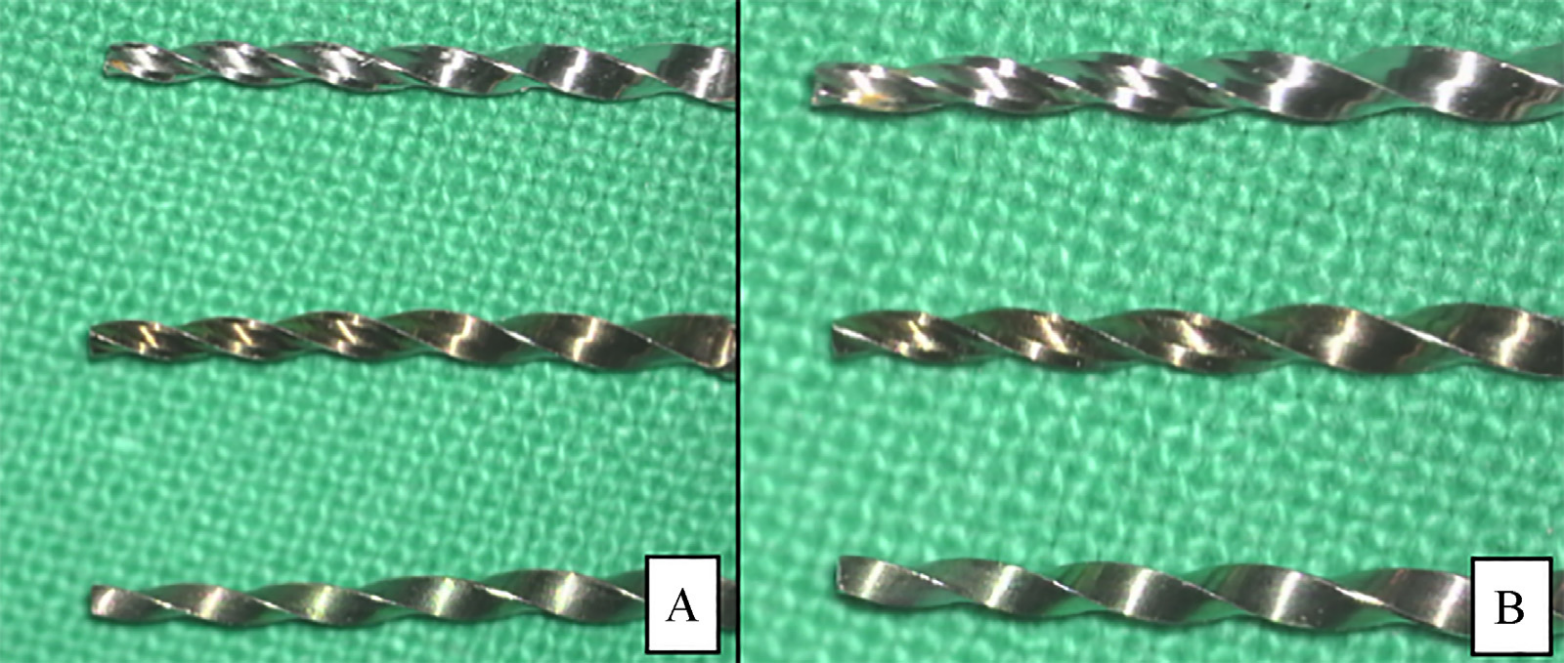
Macroscopic (20×) lateral view of the broken files using a dental operating microscope. All broken files showed a sharp break with no deformation that is a characteristic of cyclic fatigue fracture. **A**, The instruments that were tested at 20 ± 1 °C. **B**, The instruments that were tested at 37 ± 1 °C. ProTaper Universal (PTU; top), ProTaperGold (PTG; middle), and EdgeTaper Platinum (ETP; bottom).

**Fig. 3:**
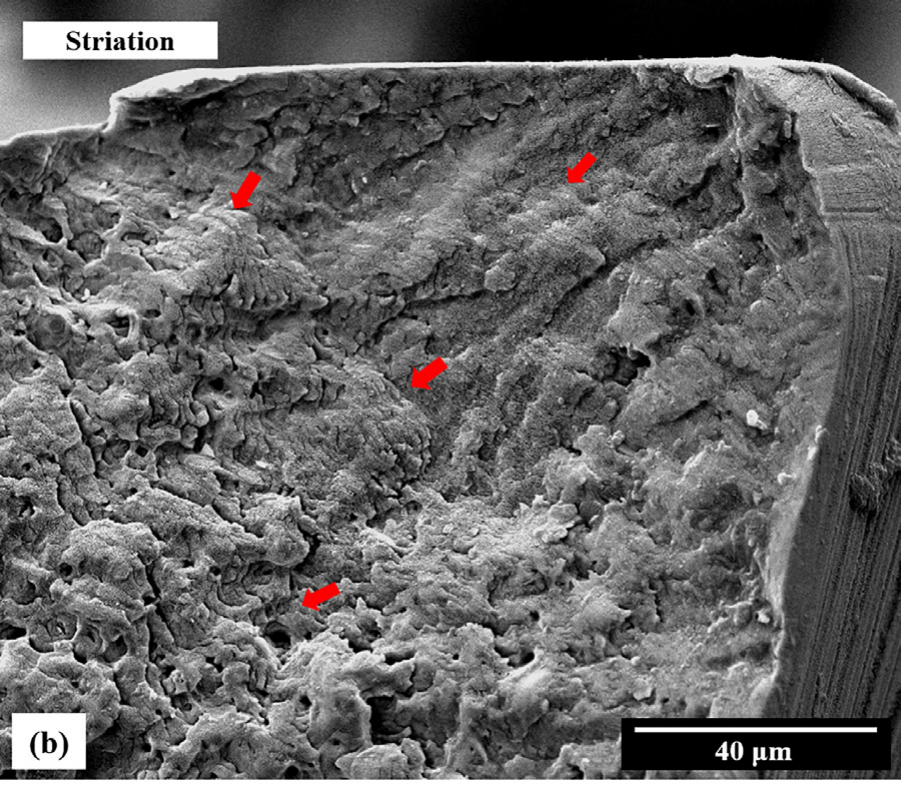
Scanning electron microscopy demonstrated evidence of fatigue failure. Red arrows indicated striation of the fracture surface on the propagation area that was markedly observed in higher magnification.

**Fig. 4:**
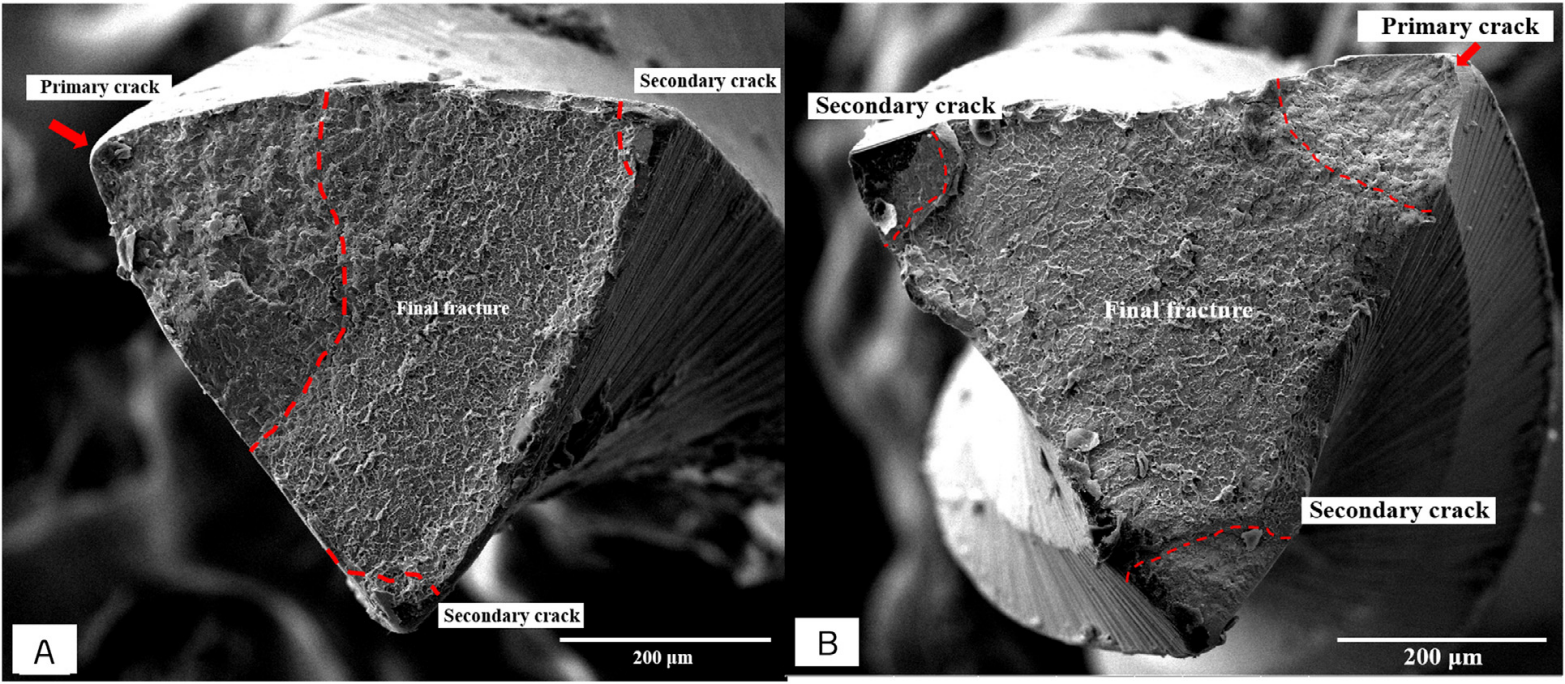
**A**, Microscopic crossectional-view of EdgeTaper platinum file using scanning electron microscope. The red dotted lines show the area of the primary and secondary cracks. **B**, Microscopic cros-sectional view of ProTaper Universal file using scanning electron microscope. The red dotted lines show the area of the primary and secondary cracks.

We evaluated the failure behaviour of the tested specimens. The lateral view macroscopic inspection of the samples revealed a sharp break (no distortion) at the fracture site (Figure 2), which demonstrated cyclic fatigue fracture characteristics. The fracture surface morphology in the microscopic investigation demonstrated cyclic fatigue behaviour that was dominated by bending behaviour (Figure 3). Furthermore, the crack propagation behaviour indicated that the ETP group allows the cracks to extend (Figure 4A), enhancing the fatigue life in service, whilst the PTG and PTU groups demonstrated shorter crack propagation (Figure 4B) and had more brittle behaviour at the final fracture surface. The failure behaviour of the files at the different tested temperatures was the same.

## Discussion

To compare the results amongst rotary file systems, the shape and size of the artificial canal block is critical. The results from testing instruments with different designs in the same artificial canal block are not comparable due to the different files’ trajectories that exhibit a different angle and radius of curvature depending on how loose the instruments are in the artificial canal. The files’ trajectories may be different between systems due to many factors (size, shape, design, pitch length, taper, and geometric features).^18^ Thus, an artificial canal block design can favour or compromise the cyclic fatigue resistance of the tested instruments. In this study, the instruments shared the same size and design and were tested in the same artificial canal block that was produced by milling technology to resemble their design; thus, the comparison amongst groups was valid.

The selection of the F5 file, whose size is greater than usual for shaping a curved canal, as a representative in each group was limited due to the milling technology (smallest milling head) available in the authors’ country. However, the instrument size had no effect on the results, because the aim of the study was focused on the type of produced alloys. Changing the tested instruments’ size may slightly affect the differences amongst groups. However, the results revealed marked differences (*P* < .001). Thus, the findings in this study can apply to every instrument produced from the tested alloys.

The cyclic fatigue resistance testing in this study was designed as a static test. Dynamic file movement may better represent the clinical situation. However, this method would be more prone to experimental errors and be more technically sensitive than a static test because a precise trajectory of the files is difficult to obtain.^19^^,^^20^ Furthermore, a static test can provide better information concerning the instrument's design or type of alloys, which was more appropriate for this study.

The type of instrument failure in this study was confirmed macroscopically and microscopically to be cyclic fatigue failure. The macroscopic lateral view of the broken files did not demonstrate any distortion or unwound flutes, which are signs of torsional failure.^5^ The microscopic investigation (Figure 4) demonstrated that the morphology of fracture surface was dominated by bending fatigue behaviour. The fatigue crack nucleation was always observed at the edge of the specimen due to the concentration of the stress field. The propagation demonstrated that the transformation induced dislocation. Furthermore, the fracture surface at the propagation area has a striation microstructure, which is evidence of fatigue failure (Figure 3). Thus, the assay setup in this study was appropriate for cyclic fatigue testing.

The functional properties of shape memory alloy are due to a reversible diffusionless phase transition, the so-called thermoelastic martensitic transformation (TMT), between two distinct crystal structures, the parent austenite (B2) and the product martensite (B19’) phases. Austenite is a relatively ordered body-centred cubic structure that is stable at high temperatures and low stresses, whereas martensite is a less ordered monoclinic phase stable at low temperatures and high stress. Thus, TMT can be activated either by temperature (thermally induced martensite [TIM]) or mechanical stresses (stress-induced martensite [SIM]). TIM and SIM both play a meaningful role in crack formation and propagation mechanisms under static and/or fatigue loadings.^21^ Cyclic fatigue resistance improvement results from a file having more martensitic microstructure.^22^ Gold-Wire alloy was affected by body temperature due to its austinite finishing (Af) temperature (50.1 ± 1.7 °C),^23^ which is higher than body temperature. Thus, at body temperature, the martensitic/austenitic ratio of the alloy was reduced. The reduced Fire-Wire alloy cyclic fatigue resistance may be due to the same reason because it is a controlled memory wire like the Gold-Wire alloy. Studies have revealed that conventional NiTi alloy (produced by Maillefer) had an Af temperature that was lower than body temperature (21.2 ± 1.9 °C^23^ and 10 °C^24^). Theoretically, this alloy should have a full austenitic microstructure at both room and body temperature. However, reduced temperature is not the only mechanism that can cause in a change from austenitic to martensitic microstructure. Another mechanism is the stress-induced martensite concept that earlier generation austenitic NiTi rotary instruments benefitted from whilst working in the root canal regardless of temperature. This concept involves the stress that is applied to the instrument whilst the instrument is operating in the root canal.^25^ The stress is caused by the root canal curvature that bends the instrument and the friction between the instrument's surface and root canal wall.^26^ Thus, one possible explanation for the decreased cyclic fatigue resistance in austenitic files is that the body temperature heat reduced the martensitic microstructure that was converted by the stress-induced martensite transformation mechanism. Furthermore, there might be other factors that are not fully understood concerning the alloy files’ reduced cyclic fatigue resistance.

The effect of temperature on the cyclic fatigue resistance results corresponded to previous studies for conventional^11^^,^^13^ and Fire-Wire^27^ alloys. However, the Gold-Wire results were different.^11^ This difference may be due to the different medias used for simulating a specific temperature. A Gold-Wire study determined the files’ cyclic fatigue resistance in a temperature-controlled oven and found that the reduced Gold-Wire cyclic fatigue resistance was not significant.^11^ In contrast, our study used distilled water as a medium to transfer the environmental temperature to the file. Liquids are a better heat transfer media compared with air. Using a liquid media may accelerate the heat transfer and generate significant results. However, the comparison between systems results corresponded to previous studies in which high temperature had a negative effect on both alloys.^11^^,^^28^

When comparing the cyclic fatigue resistance from different types of produced alloy, other factors that influence cyclic fatigue resistance need to be controlled. The PTU and PTG files have the identical geometric design in size and taper. The rotation speed, angle of file access, environmental temperature, and artificial canal used were the same.^29^ Thus, the differences in cyclic fatigue resistance resulted from only the different alloy types. The ETP files’ geometric design is indistinguishable from that of the PTU and PTG files; however, it is not identical because they are produced by a different manufacturer. This was the first study that demonstrated a difference between the F5 ETP and ProTaper Universal/Gold files from the fractured surface investigation. The ETP file cross-section is convex triangular, which is the same as ProTaper Universal/Gold in shaping (S1 and S2) and small finishing (F1 and F2) files. However, the cross section of the larger finishing files (F3, F4, and F5 in ProTaper Universal/Gold) are concave triangular to increase flexibility and improve the cyclic fatigue resistance of the instruments. Thus, the ETP files had an inferior cross-section design for cyclic fatigue resistance due to its large core mass.^6^ Notably, the cyclic fatigue resistance in the ETP group was multiple-fold greater compared with the PTU and PTG groups. Therefore, it might be concluded that the high resistance was due to the Fire-Wire alloy, as found in previous studies that investigated instruments with the same design but differed in the type of produced alloys.^8^^,^^9^^,^^11^^,^^12^

Heat treatment affected the files’ cyclic fatigue life: All 3 specimens demonstrated multiple crack nucleation at the edge of the triangle origin of the cross-section surface because of stress concentration. The PTU and PTG groups behaved similarly where the crack propagation demonstrated short extension. However, marked crack propagation extension was observed in the ETP specimen as shown by the larger crack propagation area. Therefore, this result indicated that either heat treatment in the EPT group or a different cross-section allow the extension of crack behaviour and enhance the resistance to fatigue life during cyclic loading.

Currently, there is still no standard cyclic fatigue resistance testing protocol. After all, testing at body temperature, which is similar to the actual temperature when performing root canal preparation, is more clinically relevant compared with testing at room temperature. However, the limitation of this study is that the model was set in a water environment and the artificial canal cannot replace the root canal dentin, which is a major limitation in all cyclic fatigue studies. Although simulating a single curvature does not adequately mimic the real root canal system, every simulated canal scenario is just one of the multitude of scenarios that a clinician might encounter. Care should be taken when interpreting the results of an in vitro study that does not fully simulate the real clinical situation. However, there were marked differences in NCF amongst the groups, which can be extrapolated to clinical use.

In conclusion, under the conditions of this study, all file types were affected by temperature. Cyclic fatigue resistance was reduced at higher temperature and increased at lower temperature. If the files have an identical design, the files made of Fire-Wire are preferred to Gold-Wire and conventional NiTi alloys based on cyclic fatigue resistance. Clinicians may use these data for choosing the optimum instrument in their clinical practice. Moreover, manufacturers can use our findings for future development of their file systems by choosing the appropriate produced alloy. Lowering the temperature of the files may improve their cyclic fatigue resistance.

## Conflict of interest

The authors have no competing interests related to this article.

## Appendix Supplementary materials

Image, application 1
