# Supplementary material for: Effect of Temperatures on Cyclic Fatigue Resistance of 3 Different Ni-Ti Alloy Files
Source: Int Dent J. 2023 Jul 7;73(6):904–9. doi: 10.1016/j.identj.2023.06.008 (PMC10658440; doi:10.1016/j.identj.2023.06.008)
Supplement: Supplementary file 1 [file mmc1.pdf]

Faculty of Dentistry, Chulalongkorn University

Bangkok, Thailand

May 22, 2023

Dear Dr. Lakshman Samaranayake,

This letter is to certify that I have reviewed and revised the manuscript entitled “Effect of temperatures on cyclic fatigue resistance of 3 different Ni-Ti alloy files” for English language content.

Best regards,

Kevin Tompkins DMD, PhD

Office of Research Affairs
Office of International Affairs
Mineralized Tissue Research Group
Faculty of Dentistry, Chulalongkorn University
